# A comparison of rapid weight loss practices within international, national and regional powerlifters

**DOI:** 10.1177/02601060231201892

**Published:** 2023-09-11

**Authors:** Paul Campbell, Daniel Martin, Melissa J Bargh, Thomas I Gee

**Affiliations:** 1 Department of Advanced and HE Sport, 11596 Lincoln College, Lincoln, UK; 2 School of Sport and Exercise Science, 4547 University of Lincoln, Lincoln, UK

**Keywords:** Weight-cutting, powerlifting, mood, rapid weight loss, athletes

## Abstract

**Background:** Rapid weight loss (RWL) practices are common amongst strength-sport athletes to ‘make weight’ for a chosen weight class. **Aim:** This study compared the RWL practices of International Powerlifting Federation (IPF) powerlifters from Great Britain. **Methods:** Participants (n = 69, male = 36, female = 33) were recruited from IPF lifting populations (mandatory <2-hour competition weigh-in). Participants were categorised based on highest level of competition (regional, national and international) and also sex (male and female). The previously validated ‘Rapid Weight Loss Questionnaire’ established RWL practices, however also included an open-ended question regarding thoughts, feeling and mood during RWL. **Results:** Nearly all participants (97%) had purposely acutely reduced body mass to compete, with an average typical pre-competition loss of 4.2%. Regional competitors reported a higher ‘typical’ body mass loss compared to international competitors (5.5% vs 3.3%, p = 0.004). Females reported a greater ‘highest’ body mass loss than males (6.7% vs 5.3%, p = 0.028). Fluid restriction (86.5%), water loading (67.2%) and increased exercise (49.2%) were the RWL methods most commonly used ‘always’ or ‘sometimes’. Content analysis revealed a predominance of negative perceptions during RWL with the emergence of codes; fatigue, sensations, anxiety, low mood and irritation, accounting for ∼70% of responses. **Conclusion:** Prevalence of RWL is high amongst competitive powerlifters, with many competitors attributing negative perceptions during the weight-cutting process. The magnitude of reported acute RWL within regional lifters was beyond where performance decrements are commonly seen (>5%), this should be cautioned against given the IPF's mandatory <2-hour competition weigh-in.

## Introduction

Weight class sports such as strength sports (e.g. olympic weightlifting and powerlifting) and combat sports (e.g. judo, wrestling, boxing and mixed martial arts) require athletes to compete in weight divisions, to prevent size and strength disparities (Ferland and Comtois, 2019; Reale et al., 2018). Athletes competing in such sports generally desire to have a high lean body mass and low body fat percentage to achieve a high power-to-weight ratio (Sundgot-Borgen and Garthe, 2011). Competitors are required to obtain a selected body mass during an official pre-competition weigh-in, a process known as ‘making weight’ (Matthews et al., 2019). Consequently, weight-making strategies such as voluntary rapid weight loss (RWL) have become common practice (Nolan et al., 2022; Reale et al., 2018). During competition preparation, athletes utilise a range of chronic and acute body mass loss strategies. Gradual body mass loss has been preferentially recommended to reduce body fat, minimise muscle wasting and reduce dehydration compared to RWL (Franchini et al., 2012). However, RWL has been identified as a key component of weight class sport culture and often perceived by athletes to be a fundamental aspect of competition preparation (Oppliger et al., 2003; Pettersson et al., 2013). Artioli et al. (2016) described RWL as a significant body mass reduction (2–10%) mostly within 2 to 3 days of competition weigh-in. With awareness of the availability of an rapid weight gain (RWG) period, athletes attempt to reduce glycogen levels, minimise gut content and reduce body fluid stores, whilst optimising lean body mass and minimising fatigue pre-competition (Connor and Egan, 2019; Kons et al., 2017).

RWL has been documented within strength sports. Primary federations such as the International Powerlifting Federation (IPF) and International Weightlifting Federation adopt a mandatory <2-hour weigh-in, limiting the opportunity for RWG (Nolan et al., 2022). A relatively high prevalence of pre-competition RWL has been reported within powerlifters (∼86%), with a moderate typical body mass loss (∼3%) (Kwan and Helms, 2022; Nolan et al., 2022). Wood et al. (2022) highlighted the need for further qualitative research within powerlifters to explore motivations and pressures experienced regarding RWL practices. Such research would require an inductive approach which has yet to feature within the existing body of research. Kwan and Helms (2022) did examine prevalence of five perceived psychological states within powerlifters engaging in RWL methods, although via a categorical questioning approach, rather than inductive ‘open-ended’ qualitative questioning.

The IPF is recognised as the major governing body and the largest international powerlifting association (Ball and Weidman, 2018; Travis et al., 2021). The IPF adopt a mandatory <2-hour period to re-gain body mass post-weigh-in (Moore et al., 2019). With limited time to re-gain body mass, it could be assumed that IPF powerlifters adopt less extreme body mass management practices. This notion is supported by Kwan and Helms (2022) who reported a moderate (2.9%) pre-competition typical body mass loss within IPF powerlifters. With this in consideration it is important to identify the RWL practices used by IPF powerlifters of various competitive standards. As powerlifters competing at a lower level (regional and national) would likely not have the competitive experience or access to the level of coaching and support staff compared to international level lifters (Travis et al., 2021).

The aim of this study was to describe the RWL practices of IPF powerlifters from Great Britain and to compare the RWL practices of those competing at international, national and regional level. To our knowledge this is the first study to feature open-ended questioning and subsequent inductive analysis regarding ‘strength sport’ or powerlifting athletes thoughts, feelings and mood during the weight-cutting process.

## Methods

### Participants

All participants competed within ‘British Powerlifting’ sanctioned competitions (Great Britain's IPF affiliate powerlifting organisation). Participants were recruited from both ‘Equipped’ and ‘Classic’ category lifting populations via a direct ‘face to face’ approach during competitions and at training venues. Prior to survey completion, all participants were informed of the benefits and risks of the investigation prior to signing an institutionally approved informed consent document to participate in the study. The participants were categorised and grouped based on their highest level of competition within the previous 12 months (e.g. international, national and regional) and then also separately via sex (male and female). The study was approved by the School of Sport and Exercise Science ethics committee at the University of Lincoln in accordance with Helsinki Declarations for research with human volunteers and was assigned the reference number: 2019-1031.

### Questionnaire

The survey titled ‘Pre-competition weight management practices questionnaire’ was used to establish the common body mass management practices and the magnitude of RWL across groups. This survey was slightly adapted from the previously validated ‘Rapid Weight Loss Questionnaire’ (RWLQ) (Artioli et al., 2010c), to include questions under themes such as ‘water loading’, ‘Hot water immersion’ and ‘hot salt water immersion’ to reflect current reported practices amongst athletes (Connor and Egan, 2019; Nolan et al., 2022). In addition, the survey featured an open-ended question regarding reported thoughts, feelings and general mood during the weight-cutting process. The questionnaire contained 23 questions in total.

### Data analysis

The RWLQ was scored as suggested by Artioli et al. (2010c) to produce an RWL score (RWLS). The additional questions related to themes of ‘water loading’, ‘hot water immersion’ and ‘hot salt water immersion’ were not scored in the final calculation of the RWLS. Descriptive statistics (mean ± SD) were used to display participant characteristics and responses to closed-ended survey questions. For group data across the two aspects of comparison; competitive level (international, national and regional) and sex (male and female) statistical analyses were completed using SPSS (Version 25.0, IBM Corp., Armonk, NY). The Levene's test for equality of variances and the Shapiro-Wilk test for normal distributions were performed. Following this, for competitive level comparison a Krustal-Wallis one-way analysis of variance was performed to determine any significant differences (p ≤ 0.05) between the three groups. Where a significant difference was shown, Dunn's post hoc pairwise comparison tests were carried out between groups using the Bonferroni correction. For sex comparisons an independent samples Mann-Whitney U test was performed (p ≤ 0.05). Variables analysed across groups were RWLS, ‘highest’ body mass loss before competition, ‘typical’ body mass loss and ‘typical’ body mass gain. Body mass loss and gain variables were analysed in relative terms (percentage of body mass) for comparisons across competitive levels and sex.

### Qualitative data analysis

The qualitative component of the questionnaire was analysed according to principles for content analysis (Miles and Huberman, 1994) and thematic analysis (Clarke and Braun, 2017). The authors first enhanced familiarity with the data by reading and re-reading each qualitative component of the questionnaire (Maykut and Morehouse, 1994). Then, adopting an inductive approach, *codes* were generated, by identifying relevant extracts of data from the qualitative aspect of the questionnaire. These codes were then combined into categories based on the frequency of occurrence of keywords to capture the wider meaning of the data and to illustrate areas of consistency in the responses. Subsequent to these steps, a ‘critical friend’ approach resulted in changes to some codes and categories, which were critically challenged by the authors to reduce the potential for error and misinterpretation of the data.

## Results

### Sample characteristics

The sample consisted of 69 responses (male n = 36; female n = 33), including 43 ‘Classic’ and 26 ‘Equipped’ powerlifters. Male and female weight class frequency distribution was as follows (males: 59kg = 1, 66kg = 3, 74kg = 7, 83kg = 2, 93kg = 12, 105kg = 8, 120kg = 3; and females: 47kg = 1, 52kg = 5, 57kg = 6, 63kg = 9, 72kg = 8, 84kg = 4). Mean age of the sample was 29.7 ± 7.4 years, mean competitive experience was 5.7 ± 5.9 years, mean standard based on ‘IPF Good lift points’ was 82.6 ± 11.9 points. Within 12 months prior to completing the survey, with regards to the highest level of competition participated, 22 participants had competed at an international level, 32 at a national level and 15 at a regional level. The mean number of competitions participated in was 3.4 ± 1.7 within 12 months prior to completing the survey.

### Pre- and post-weigh-in body mass loss and re-gain

Of the total responses, 67 of 69 (97%) of participants surveyed had previously purposely acutely reduced body mass to compete. All subsequent data communication will be in consideration of these 67 participants (Table 1). The most common time allocated to cut body mass before competition for the sample was 0 to 7 days (n = 28, 42%), followed by 8 to 14 days (n = 23, 34%), then 15 days and over (n = 16, 24%). The mean ‘typical’ acute body mass loss leading into competition was 4.2 ± 1.9%. However, mean ‘highest’ career weight cut was 5.9 ± 2.5% of body mass. In addition, There was a significant difference in ‘typical’ body mass between competitive levels (H_(2)_ = 10.288, p = 0.006). Body mass loss was higher in regional (5.5 ± 2.2%) than international competitors (3.3 ± 1.5%, p = 0.004). There was no difference in ‘typical’ or ‘highest’ body mass loss between international and national competitors (typical; p = 0.316, highest; p = 0.270). There was no difference in ‘typical’ body mass loss between males and females (U = 617.000, p = 0.474). However, females reported a significantly greater ‘highest’ body mass loss than males (6.7 ± 2.6% vs 5.3 ± 2.2%, U = 384.500, p = 0.028).

**Table 1.** Descriptive characteristics and weight loss and weight gain data of weight-cutting IPF powerlifters (n = 67) and as grouped based on sex and competitive level.

	All (n = 67)	Male (n = 35)	Female (n = 32)	International (n = 21)	National (n = 32)	Regional (n = 14)
Age (y)	29.8 ± 7.4	28.7 ± 7.0	31.0 ± 7.9	31.9 ± 7.8	29.5 ± 7.7	27.4 ± 5.4
Body mass (kg)	80.1 ± 19.3	92.9 ± 16.7	66.1 ± 10.1	72.6 ± 17.0	81.5 ± 21.4	88.1 ± 14.2
Height (m)	1.69 ± 0.10	1.75 ± 0.08	1.62 ± 0.07	1.65 ± 0.10	1.68 ± 0.11	1.76 ± 0.06
Mean competitive experience (year)	5.7 ± 6.0	6.5 ± 7.2	4.9 ± 4.1	7.8 ± 6.8	5.2 ± 6.2	4.0 ± 2.7
IPF good lift points	82.5 ± 12.1	82.6 ± 12.1	82.5 ± 12.2	89.3 ± 13.5	80.7 ± 10.2	76.4 ± 9.2
RWL score	23.7 ± 8.1	25.3 ± 7.9	22.0 ± 7.9	21.7 ± 6.1	24.0 ± 9.4	25.9 ± 6.9
Number of competitions in past 12 months	3.4 ± 1.7	3.1 ± 1.6	3.9 ± 1.8	4.4 ± 1.6	3.4 ± 1.7	2.0 ± 0.7
Number of competition weight cuts in past 12 months (and % of competitions)	2.4 ± 1.5 (70.6%)	2.1 ± 1.2 (67.7%)	2.8 ± 1.7 (71.8%)	2.8 ± 1.4 (63.6%)	2.5 ± 1.7 (73.5%)	1.6 ± 0.6 (80%)
Highest ever cut to compete (body mass %) (and kg)	5.9 ± 2.5% (4.7 ± 2.1kg)	5.3 ± 2.2% (4.9 ± 2.2kg)	6.7 ± 2.6%* (4.5 ± 2.1kg)	5.3 ± 2.5% (3.8 ± 1.8kg)	6.2 ± 2.7% (4.9 ± 2.3kg)	6.2 ± 1.8% (5.5 ± 1.8kg)
Typical competition weight cut (body mass %) (and kg)	4.2 ± 1.9% (3.3 ± 1.8kg)	4.3 ± 2.0% (3.9 ± 1.9kg)	4.0 ± 1.9% (2.7 ± 1.5kg)	3.3 ± 1.5% (2.4 ± 1.0kg)	4.1 ± 1.8% (3.3 ± 1.6kg)	5.5 ± 2.2%** 4.9 ± 2.2kg
Typical weight re-gain in week after competition (body mass %) (and kg)	3.8 ± 2.2% (3.0 ± 1.9kg)	4.3 ± 2.4% (3.8 ± 2.0kg)	3.4 ± 1.9% (2.2 ± 1.3kg)	3.1 ± 1.6% (2.2 ± 1.1kg)	3.7 ± 1.9% (2.9 ± 1.6kg)	5.3 ± 2.9% (4.5 ± 2.6kg)

*Significant difference compared to male (p < 0.05).

**Significant difference compared to international (p < 0.05).

IPF: International Powerlifting Federation; RWL: Rapid weight loss.

Reported ‘typical’ body mass gain 1-week post-competition for the whole sample was 3.8 ± 2.2% of body mass. There were no significant differences in body mass gain between competitive groups (H_(2)_ = 5.831, p = 0.054), despite a mean body mass gain of 5.3 ± 2.9% in regional competitors compared to national (3.7 ± 1.9%) and international competitors (3.1 ± 1.6%). There was no difference in body mass gain between males and females (U = 695.500, p = 0.089). The mean RWLS of sample was 23.7 ± 8.1, with no significant differences across competitive groups (H_(2)_ = 2.773; p = 0.250) or sex (U = 704.000, p = 0.071).

### Rapid weight loss methods used and source of influence

For RWL methods used (Table 2), frequencies of ‘always’ were reported highest for ‘fluid restriction’ (55.2%), ‘water loading’ (40.3%) and ‘Fasting (not eating all day)’ (13.4%). A high proportion of athletes (55.2%) reported ‘always’ using ‘Gradual dieting to lose weight in 2 weeks or more’. Passive heat methods such as ‘hot water immersion’, ‘hot salt water immersion and ‘Saunas’ were used less frequently (‘always’ = 1.5–7.5%), data indicated that these methods received occasional use within the cohort (‘sometimes’ = 13.4–31.3%). Sources who were ‘Very influential’ and/or offered ‘some influence’ to body mass loss practices were athletes within the same sport (61.2%), coaches (55.2%) and internet sources (46.2%; Table 3). Sources rated as ‘Not influential’ were ‘Doctors’ (97%), ‘Parents’ (95.5%) and ‘Personal trainers’ (92.5%).

**Table 2.** Frequency analysis of RWL methods used by IPF powerlifters in Great Britain, values expressed as a percentage of responses reported to each category.

Method	Always	Sometimes	Almost never	Never used	No longer use
Gradual dieting over 2 weeks or more	55.2	35.8	0.0	7.5	1.5
Skipping 1 or 2 meals	11.9	32.8	14.9	35.8	4.5
Fasting (not eating all day)	13.4	16.4	10.4	55.2	4.5
Restricting fluid ingestion	55.2	31.3	1.5	9.0	3.0
Increased exercise (more than usual)	11.9	37.3	11.9	35.8	3.0
Training intentionally in heated training rooms	1.5	0.0	4.5	91	3.0
Saunas	7.5	13.4	7.5	62.7	9.0
Hot water immersion	6.0	31.3	10.4	46.3	6.0
Hot salt water immersion	1.5	14.9	7.5	70.1	6.0
Training with rubber/plastic layers	0.0	1.5	0.0	95.5	3.0
Use rubber/plastic layers without exercise	1.5	4.5	3.0	88.1	3.0
Spitting	3.0	13.4	19.4	59.7	4.5
Water loading	40.3	26.9	11.9	16.4	4.5
Laxatives	4.5	11.9	3	74.6	6.0
Natural diuretic supplement	3.0	13.4	7.5	73.1	3.0
Diet pills	0.0	0.0	3.0	95.5	1.5
Vomiting	0.0	3.0	1.5	94	1.5
Other (please state)	11.9	3.0	0.00	85.1	0.0

IPF: International Powerlifting Federation; RWL: Rapid weight loss.

**Table 3.** Frequency analysis of the sources of influence on the weight-making practices of IPF powerlifters, values expressed as a percentage of responses reported to each category.

Source	Very influential	Some influence	Unsure	Little influence	Not influential
Nutritionist	20.9	9.0	3.0	3.0	64.2
Doctor	3.0	0.0	0.0	0.0	97.0
Personal trainer	0.0	3.0	3.0	1.5	92.5
Parent	0.0	0.0	1.5	3.0	95.5
Coach	23.9	31.3	1.5	10.4	32.8
Other athletes (same sport)	19.4	41.8	4.5	7.5	26.9
Other athletes (different sport)	3.0	22.4	6.0	6.0	62.7
Published journal articles	11.9	20.9	7.5	10.4	49.3
Book/magazine	4.5	3.0	6.0	7.5	79.1
internet sources	14.9	31.3	6.0	11.9	35.8

IPF: International Powerlifting Federation.

### Qualitative findings

In regards to the open-ended question ‘*Please describe your main thoughts, feelings and general mood during the weight cutting process’*, 66 of 67 weight-cutting participants provided an answer. Responses were analysed in accordance with content analysis into three key categories. Table 4 depicts these example quotes, codes, categories and the total number and percentage of participants whose responses made up the categories.

**Table 4.** Participants’ cognitions, physiological perceptions and emotional feelings and mood during the weight-cutting process.

Example quotes	Code (frequency; percentage)	Category
‘Energy is lower as low-calorie intake, feeling restricted and limited in food options can significantly affect training.’ ‘Day before and morning of comp water is cut, feel ‘out of it’ tired and drained.’ ‘Drained, fatigued, tired, hungry, skinny, weak.’	Fatigue (15; 23%) International (2; 10%) National (7; 22%) Regional (6; 43%) Male = (9; 26%) Female = (6; 19%)	Physiological perceptions
‘Very dehydrated when cutting out water after water loading.’ ‘Occasionally I get cramps, presumably due to lowering salt.’ ‘Headache and thirst during water cut.’	Sensations (4; 6%) International (0; 0%) National (4; 13%) Regional (0; 0%) Male (4; 11%) Female (0; 0%)	
‘Cleaning up my diet is part of my prep and gets my head in a good focus for competing.’ ‘Thoughts of planning and following the plan for weight cutting. Visualising what I’m going to hit on the platform.’ ‘Generally focused during the weight cut.’	Focus (11; 17%) International (7; 35%) National (4; 13%) Regional (0; 0%) Male (5; 14%) Female (6; 19%)	Cognitions
‘Main anxiety comes from not trusting my scales at home to be the same as the competition scales, energy expenditure goes down during peak/taper so I worry weight will go up.’ ‘Anxious about not making weight’. ‘Do not enjoy the process, that's why I also moved up a weight category, I’m an anxious person and it was not worth it.’ ‘Sometimes felt it mentally hard and because I’m food obsessed, however recently I have worked with a nutrition coach and have found it much more positive.’ ‘Quite like it. See it as a challenge. Helps get into competition mindset’. ‘First week hard to cut out the treats.’	Anxiety (9; 14%) International (4; 20%) National (4; 13%) Regional (1; 7%) Male (3; 9%) Female (6; 19%) Challenge (3; 5%) International (0; 0%) National (2; 6%) Regional (1; 7%) Male (2; 6%) Female (1; 3%)	
‘I feel pretty sad as there is a restriction in food or fluid has to increase.’ ‘I was low, I felt disgusting. I am never going to restrict myself the way I have done ever again’. ‘Last 3 days are miserable. Very strict. Low on fasted diet with no training. Low mood agitated.’	Low mood (10; 15%) International (0; 0%) National (7; 22%) Regional (3; 21%) Male (4; 11%) Female (6; 19%)	Emotional responses
‘Get more irritable quickly when restricting food intake.’ ‘I get more emotional and irritated.’ ‘I do find it frustrating in the week before, especially watching my family eat anything.’	Irritation (8; 12%) International (4; 20%) National (2; 6%) Regional (2; 14%) Male (4; 11%) Female (4; 13%)	
‘Positive during process as weight loss can be achieved with minimal changes to diet and water manipulation.’ ‘Pretty good. Stay positive and trust the process.’ ‘My last cut was with a nutritionist who was excellent. I felt amazing apart from some occasional frustration at having to miss out on take always etc.’	Positive mood (6; 9%) International (3; 15%) National (2; 6%) Regional (1; 7%) Male (4; 11%) Female (2; 6%)	

*Note:* Total numbers of participants for each competitive level are outlined as follows: international (20), national (32), regional (14); male (35) and female (31).

## Discussion

The study identifies that a high proportion of powerlifters (97%) practice acute pre-competition body mass loss in an attempt to ‘make weight’. The level of prevalence was in line with cohorts of combat athletes (judo, amateur boxing and kickboxing) involved in ‘<2-hour’ or ‘day of competition’ weigh-ins (90–100% pre-competition RWL prevalence) (Dugonjić et al., 2019; Kons et al., 2017; Reale et al., 2018). Our prevalence rate (97%) is equal to that reported with mixed martial arts (MMA) fighters who were subjected to weigh-ins on the day before (24–36 hours) competition (Connor and Egan, 2019). Indeed, this proportion is greater than the prevalence of weight-cutting as reported within previous cohorts of competitive powerlifters (44–86%) (Kwan and Helms, 2022; Nolan et al., 2022; Wood et al., 2022).

The magnitude of typical pre-competition body mass loss (4.2% of body mass) was also higher than as previously reported within powerlifters (∼3.0%) (Kwan and Helms, 2022; Nolan et al., 2022). Within our study the most common time allocated to acutely reduce body mass before competition was 0 to 7 days (42%) followed by 8 to 14 days (34%), with 24% beginning body mass loss over a longer period of 15 days and over. Comparable RWL studies within powerlifters do not report time allocation data (Kwan and Helms, 2022; Nolan et al., 2022). However, Wood et al. (2022) reported that only 1 and 5 participants of a cohort of 37, undertook RWL practices less than 1 week and 1 to 2 weeks, respectively, before competition, with the majority of participants engaging in longer duration pre-competition body mass reduction strategies (19 of 37; 6–8 weeks prior to competition). Within cohorts of combat athletes, subjected to on the day competition weigh-ins, mean number of days to cut body mass has been recorded to be ∼10 to 17 (Dugonjić et al., 2019; Reale et al., 2018). Although, Artioli et al. (2010a) reported that 72% of Judo athletes were engaged in the weight-cutting process 0 to 7 days before competition.

The RWLS in the current study was 23.7, similar to what was reported within drug-tested powerlifters (25.1), who were subjected to <2-hour competition weigh-in (Nolan et al., 2022). Although higher RWLS have been documented within IPF powerlifters (31.5, Kwan and Helms, 2022) and drug-tested powerlifters subjected to 24-hour pre-competition weigh-ins (31.1; supplemental data provided by Nolan et al., 2022). Higher RWLS have been reported within a range of combat sport athlete cohorts adopting <2-hour weigh-ins (29.5–31.7) (Reale et al., 2018). Although, this is unsurprising given the longitudinal established culture of weight cutting within combat sports (Barley et al., 2019). Considerably higher RWLS (37.9) have been reported in combat sport populations with pre-competition weigh-in windows exceeding 24 hours, which co-existed with athletes undertaking a considerable mean typical pre-weigh-in body mass loss (7.9% body mass) (Connor and Egan, 2019).

Regarding acute RWL methods listed as ‘always’ used by athletes ‘gradual dieting to lose weight in 2 weeks or more’, ‘restricting fluid ingestion’ and ‘water loading’ were most commonly reported (40.3–55.2%), these findings are in accordance with previous cohorts of powerlifters (33–54% attributing ‘always’ used for these methods) (Kwan and Helms, 2022; Nolan et al., 2022; Wood et al., 2022). Passive methods that induce RWL via increases in body temperature and sweating (sauna, hot water immersion and hot salt water immersion) were used occasionally by participants, such as hot water, being ‘always’ or ‘sometimes’ used by 37.3% of the cohort, with similar findings being reported within previous cohorts of powerlifters (Kwan and Helms, 2022; Nolan et al., 2022). Interestingly, these methods were rarely ‘always’ used by participants (1.5–7.5%), indicating that their use may be supplementary to the use of prevalent RWL techniques when additional body mass reductions are required. Investigations featuring athletes subjected to longer pre-competition weigh-in periods (24–36 hours), have reported much higher use of such passive heat RWL methods (Connor and Egan, 2019; Hillier et al., 2019). For example, Connor and Egan (2019) reported that 27.6% and 34.5% of MMA fighters sampled indicated ‘always’ using ‘sauna’ and ‘hot salt baths’, respectively. Sources who were ‘very influential’ or offered ‘some influence’ to body mass loss practices were athletes within the same sport (61.2%), coaches (55.2%) and internet sources (46.2%). These were also the three top ranking sources as reported by Nolan et al. (2022) within powerlifters. Interestingly, 20.3% of our cohort reported ‘nutritionist’ as a ‘very influential’ source. Indicating that regulated advice regarding RWL is deemed as impactful to those athletes who utilise it.

Within the current study, regional competitors were shown to typically cut significantly more body mass than international level competitors (5.5% vs 3.3%). There were no significant differences in typical pre-competition body mass loss between males (4.3%) and females (4.0%). These findings are in accordance with Nolan et al. (2022) who reported similar magnitudes of typical body mass loss across males and females (3.0–3.1%). However, there was significantly greater highest relative body mass loss within females (6.7% vs 5.3%). Acute body mass losses of >5% have been cautioned against particularly when associated with short recovery windows before competition, such as sports subjected to <2-hour pre-competitive weigh-in procedures (Barley et al., 2018; Franchini et al., 2012; Reale et al., 2017). In-spite of recommendations, reported performance effects following ∼5% acute body mass losses have been mixed, with some authors reporting decreases in physical performance (Barley et al., 2018, Ceylan et al. 2022, Hall and Lane, 2001), whereas others reported no effects (Artioli et al., 2010b; Mendes et al., 2013; Yang et al., 2018). However, the majority of interventions reporting no effects have utilised prolonged (>4 hours) post-weigh-in nutritional recovery windows. Decrements in performance have been shown via studies monitoring performance during shorter recovery windows (<3 hours) (Barley et al., 2018, Ceylan et al., 2022, Hall and Lane, 2001). In addition, acute body mass losses of 5% have been shown to negatively impact aspects of physical health (microcirculation and prolonged dehydration) and mood state (Ceylan et al., 2022; Hall and Lane, 2001; Yang et al., 2015). Since the contribution from dehydration would commonly substantiate a significant proportion of acute body mass losses over 5% (Barley et al., 2019; Reale et al., 2017), achieving adequate hydration status within a short post-weigh-in recovery window (<2 hours) would be often unrealistic following such an induced acute body mass loss (Barley et al., 2019; Durguerian et al., 2016). Furthermore, such efforts to rapidly rehydrate with large volumes of fluid can cause gastrointestinal discomfort, which quite possibly would be a greater detriment to performance than dehydration (Reale et al., 2017).

This was the first study to feature open-ended questioning regarding powerlifters’ thoughts, feelings and mood during the weight-cutting process. From our inductive analysis, three categories emerged regarding participants responses: physiological perceptions, cognitions and emotional responses. Of the 66 collated responses, there was a predominance of negative perceptions in codes such as fatigue, sensations, anxiety, low mood and irritation, accounting for 46 of the total responses. However, a lower proportion of the participants’ responses communicated ‘focus’ and ‘positive mood’ during weight cutting. These more positive responses are indicative of a goal-oriented approach towards pre-competition body mass loss (Pettersson et al., 2012). The findings agree with Kwan and Helms (2022) who reported that 61% and 50% of powerlifters reported that they ‘always’ or ‘sometimes’ experienced ‘fatigue’ and ‘anxiety’ respectively during weight cutting. Interestingly, the Kwan and Helms (2022) featured only negative psychological states (fatigue, anger, anxiety, isolation and depression) within their categorical questioning. However, as we have shown a substantial proportion of our cohort reported more positive responses/states. Interestingly, ‘fatigue’-related responses were proportionally higher across regional competitors (43%) compared to national (22%) and international (10%), conversely ‘focus’-related responses were higher in international competitors (35%) compared to national (13%) and regional (0%). Theoretically, these findings are indicative of the significantly higher RWL within the regional athletes and duration of competitive experience within the international athletes. States of ‘fatigue’, ‘general well-being’ and ‘emotional stress’ as assessed through profile of mood states (POMS) and recovery-stress questionnaire for athletes (REST-Q) were shown to be significantly negatively affected within olympic weightlifters who purposely reduced body mass over 6 days pre-simulated competition, compared to a group who maintained body mass over the same time period (Durguerian et al., 2016). In addition, ‘global recovery score’ was decreased pre to post in the body mass loss group, although acute weightlifting performance was not affected. The various negative mood effects experienced by participants during pre-competition RWL in the current study and those within Kwan and Helms (2022) are concerning and should be considered by powerlifters and coaches. Indeed, it has been recognised that the ability to produce appropriate emotional feeling before competition is one of the most important factors contributing to athletic performance (Durguerian et al., 2016).

## Conclusion

The prevalence of RWL is high amongst competitive powerlifters across regional, national and international levels. Regional competitors demonstrated a significantly higher typical pre-competition body mass loss. The magnitude of typical and highest reported RWL within regional lifters and highest RWL within female lifters was beyond the level where performance decrements are commonly seen with acute RWL (>5% body mass). Accordingly, such levels of RWL should be cautioned against within IPF powerlifters given the sport's mandatory <2-hour competition weigh-in. Common acute RWL methods included fluid restriction, water loading and increased exercise. Obtaining nutritional advice from an appropriately certified professional may support body mass management post-competition and prevent potentially detrimental levels of RWL leading into competitions.
